# Psychological Factors Explaining the Referral Behavior of Iranian Family Physicians

**DOI:** 10.5812/ircmj.13395

**Published:** 2014-04-05

**Authors:** Bahram Mohaghegh, Hesam Seyedin, Arash Rashidian, Hamid Ravaghi, Nader Khalesi, Hossein Kazemeini

**Affiliations:** 1 School of Health Management and Information Sciences, Iran University of Medical Sciences, Iranian Ministry of Health and Medical Education, Tehran, IR Iran; 2 Health Management and Economic Research Center, School of Health Management and Information Sciences, Iran University of Medical Sciences, Iranian Ministry of Health and Medical Education, Tehran, IR Iran; 3 Department of Health Management and Economics, School of Public Health, Tehran University of Medical Sciences, Tehran, IR Iran; 4 Ministry of Health and Education of Iran, Tehran, IR Iran

**Keywords:** Physicians, Primary Care, Referral and Consultation, Iran

## Abstract

**Background:**

The recently developed policy of the family practice program in rural regions of Iran faced some challenges such as inefficient referral system. The health insurance organizations (purchaser) and health policy makers are concerned about the high rate of patient referrals from family physicians to specialists due to imposing unnecessary services and costs.

**Objectives:**

This study examined utility of the theory of planned behavior to explain intention of Iranian family physicians to reduce referral rate of patients with respiratory diseases to medical specialist.

**Patients and Methods:**

An exploratory cross-sectional study, employing a correlational design directed by the theory of planned behavior was conducted. A questionnaire was developed based on an eliciting study and review of literature. One hundred and seventy-four family physicians working at primary care centers in two provinces of Iran completed the questionnaire (response rate of 86%).

**Results:**

The finding revealed that intention of family physicians to reduce referral rate of patients to specialists was significantly related to two theory-based variables of subjective norms (r = 0.38, P < 0.001) and perceived behavioral control (r = 0.43, P < 0.001), and not to attitudes. A stepwise regression entering direct measures of the theory variables explained 35% of the variance on the intention, with perceived behavioral control being the strongest predictor. Adding background variables to the model achieved further 5% by variables of practice size and past referral rate behavior.

**Conclusions:**

The results indicated that psychological variables of the theory of planned behavior could explain a noticeable proportion of variance in family physician's intention to decrease the rate of referring patients with respiratory diseases to medical specialists. The intention is primarily influenced by normative and control considerations. These findings contribute to a better understanding of referral decisions by family physicians and are of great value in developing interventions to reduce the variation in referral rate of patients to medical specialists at primary care health centers.

## 1. Background

The Iranian health system has experienced a number of reforms such as the Primary Health Care (PHC) expansion in 1984. PHC was aimed to achieve better health for population by providing accessible, comprehensive, and equitable health care. This initiative, despite its success to improve the Iranian health indices in following years (1, 2), faced with some challenges such as inefficient referral system (3, 4). In response to such issues, the government introduced two new policies including the family medicine program and a health insurance initiative, namely "Behbar", covering all families inhabitant in rural regions and cities with a population of less than 20000, in 2005 (5). The remuneration system of the family medicine program is based on a purchaser-provider relationship. A Public health insurance organization (purchaser) pays the District Health Centers for providing a defined PHC package through the capitation model.

In this program, general practitioners (GPs) act as Family Physicians (FPs) and provide primary cares to a defined block of population. The FPs play a gatekeeper role as they control the referral of patients to secondary care. A referral is often a dynamic process that involves multiple stakeholders and a wide range of factors influence it (6). The literature investigating factors influencing the referral pattern of GPs mostly have examined the characteristics of patients, organizations, and physicians (7-11). The contribution of patients' and both physician and practice's characteristics in explaining the variance in the referral rates have been less than 40 and ten percent, respectively (12). Thus, a part of variations in the referral rates is left unexplained (13). This could represent inappropriateness in managing patients attending the primary care centers (PCCs) and it could results in considerable implications for patients and health care system. In the context of family medicine program, the purchaser has expressed its concerns relating to the high referral rates of patients to the medical specialists. They believe that this issue is against the primary goal to reduce the unnecessary costs. In response, the purchaser has set a strict threshold limit for referral rates (i.e. 10%-12%). In cases of exceeding this level, the provider is questioned and fined if necessary. On the other hand, inappropriate referrals and variation in referral rates by GPs are concerns of policy makers (6, 12, 14).

### 1.1. Theoretical Framework

**Figure 1.**
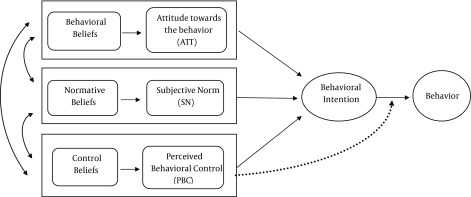
Theory of Planned Behavior (20)

In this study, the Theory of Planned Behavior (TPB) (15) is used as a theoretical framework to explain the referral behavior of FPs. This model is commonly used to explore the determinants of professional behavior (16-18). Psychological theories have successfully explained the factors influencing clinical behaviors as well as predicting behaviors in other settings (16, 18, 19). The TPB presents a model concerning how intentional human behaviors are guided (15). Based on this model (Figure 1), a behavior could be predicted by intention to perform it, perceived facilitating, and impeding factors, namely perceived behavior control (PBC). The intention itself is influenced by three cognitive constructs including attitudes (ATT), subjective norms (SN), and PBC. These variables, in turn, are based on salient beliefs about the behavior.

The behavior under investigation was specified according to the Target, Action, Context, and Time (TACT) elements (15). Outpatients presenting with respiratory disease were target population and the action was the referral decisions made by FPs. The PCCs were taken into account as context and the time scale was the following three months.

### 1.2. Clinical Context

The respiratory diseases were considered as the tracer topic for assessing the validity of the TPB in explaining the FPs' referral behavior. We followed a process to take into account some considerations to define an appropriate topic to probe the efficiency of TPB model in explaining the factors affecting the referral behavior (Appendix 1).

## 2. Objectives

In this study, we focused on the general perceived concerns of high rate of patient referrals to the medical specialists; in addition, we aimed to explore the relationship between the TPB constructs and the FPs' intention to decrease the referral rate of outpatients with respiratory diseases.

## 3. Patients and Methods

### 3.1. Design and Participants

This study was an exploratory study using TPB theoretical variables and an outcome (i.e. intention), measured by a single postal questionnaire. It was conducted between August 2012 and January 2013 at PCCs affiliated with two provincial medical universities of Lorestan and Markazi. These provinces are located in center and southwest of Iran. The target respondent population were all FPs practicing at PCCs. The participants with less than two months work experience as FP were excluded. The ethics approval was obtained by Local University Research Ethics Committee.

### 3.2. Elicitation Study

Following the TPB literature (20, 21), an elicitation study was conducted to explore the commonly held beliefs of FPs regarding to the referral behavior. A sample of 50 FPs from selected regions was randomly recruited. The salient beliefs about the topic were collected by a questionnaire using open-ended questions. The content analysis was carried out to list the most frequently stated beliefs in terms of modal salient outcomes, referents, and control factors relating to the topic. These beliefs were used to the construction of the belief items in the final TPB questionnaire.

### 3.3. Questionnaire Development

The items included in the TPB questionnaire were initially derived from previously recommended scales by Ajzen (20), Francis et al. (21), as well as from the eliciting study. The TPB questionnaire sought data on the following measures (Table 1). The outcome variable was the behavioral Intention. It comprised of three items: "I intend/I plan/the probability /to reduce the rate of referring patients with respiratory diseases to specialists in the next three months". The mean composite score of these items was taken as the intention measure. Direct and indirect measures were employed to assess the predictive variables of TPB model. The scales to assess the direct measures of ATT, SN, and PBC consisted of three to four items. The indirect measures of three mentioned variables were corresponding underlying beliefs, namely behavioral beliefs, normative beliefs, and control beliefs.

Four items assessed behavioral beliefs about positive consequences of referring patients with respiratory diseases to medical specialists. There were also corresponding items to evaluate the consequences. Five items assessed normative beliefs of FPs regarding the decisions made to refer patients. There were also five corresponding items showing respondents' motivation to comply with referent individuals or groups (e.g. Medical specialists, patients, and insurance organizations). Four items assessed control beliefs concerning referring the patients to the medical specialists. There were also four items reflecting the respondents' beliefs in regard to the perceived power of each control beliefs, which facilitate or inhibit referring patients to the medical specialists. All items were scored in a unipolar fashion from one to seven with higher number representing greater probability or favorability. The final questionnaire also included basic characteristic data regarding the respondents and practices (i.e. age, sex, respondents' experience as GP and as FP, average daily list size of consultations, practice size, and past referral rates). The main body of this self-reported questionnaire consisted of a set of 39 items including direct and indirect measures (Table 1).

**Table 1.** The Components of Developed Theory of Planned Behavior Questionnaire

Variables	Items	Score
**Backgrounds**	Participants and practice characteristics	
**Behavioral Intention**	3 items	mean of item scores
**Attitudes**		
Direct attitude	3 items	mean of item scores
Indirect attitude	8 items	a composite score formed by summing the 4 behavioral beliefs multiply 4 relevant outcome evaluation
**Subjective norms**		
Direct subjective norms	3 items	mean of item scores
Indirect subjective norms	10 items	a composite score formed by summing 5 normative beliefs multiply 5 relevant motivation to comply
**Perceived behavioral control**		
Direct perceived control	4 items	mean of item scores
Indirect perceived control	8 items	a composite score formed by summing 4 control beliefs strength multiply 4 relevant control belief power

#### 3.3.1. Validity and Reliability of the Theory of Planned Behavior Questionnaire

The face and content validity of the initial draft of the questionnaire was determined by an expert panel (including a psychologist, three members of the research team, and an internal medicine specialist). We conducted a pilot study with five FPs of non-selected statistical population that resulted in a minor revision to the questionnaire. To assess the reliability of TPB questionnaire, the criterion of temporal stability using the test-retest method was applied. In addition, we examined the internal consistency of direct measures by computing their Cronbach's alpha coefficients (15). The final version of the questionnaire was completed by 25 FPs (from a similar non-selected region) at periods two weeks apart. The test-retest reliability analysis revealed that the correlation alpha for all items was greater than 0.60 (ranging from 0.62 to 0.96). The direct measures, regardless the behavioral intention (Cronbach's alpha = 0.77), could not achieve the acceptable internal consistency (Cronbach's alpha > 0.6). We improved this criterion by deleting an item from each one.

### 3.4. Sampling

In total, 214 FPs worked in the PCCs affiliated with the two medical universities of Lorestan and Markazi. A previous methodological study conducted by Rashidian et al. (22) recommended that a minimum sample size of 148 subjects would be needed to conduct the regression analyses required in TPB studies. We used the census strategy. From the 214 FPs, seven were excluded because they did not meet the inclusion criteria of at least two months experience as a family physician. Considering a response rate of about 75 percent, we sent the questionnaires to all 207 FPs that met the inclusion criteria.

### 3.5. Data Collection Procedures

Data was collected by postal questionnaire survey. The mailings included a letter explained the purpose of study, a statement to inform the participants that their participation were voluntary, and the TPB questionnaire attached with an instruction saying how to complete it. Anonymity of all participants was emphasized practically by excluding any identification data of respondents. The non-responders were reminded twice at a two-week interval.

### 3.6. Data Analysis

We analyzed questionnaire survey data using SPSS version 18 (SPSS Inc, Chicago, Illinois, USA). The items, which negatively worded, were re-coded. Summary measures were calculated for constructs of intention, ATT, SN, and PBC from the means of the contributing items. We computed composite scores for three belief-based measures of TPB in accordance with Francis et al. (21) instruction. In the case of missing items, the mean of items in the scale was replaced. Descriptive statistics were generated to summarize the characteristics of respondents and their survey responses. The internal consistency of the direct measures was assessed using Cronbach's alpha coefficient. Bivariate associations were calculated using the Pearson chi-squared tests. Multiple regression analyses were used to examine the predictive value of TPB and background variables to explain the variation in intention. 

## 4. Results

Overall, 178 questionnaires were returned by FPs showing a response rate of 86%. After discarding four incomplete questionnaires, 174 questionnaires were analyzed. Internal consistency of the TPB direct measures was satisfactory for intention (r = 0.83) and ATT (r = 0.75), but not for SN and PBC. To address this, one item from each unsatisfied variable was removed; it resulted in achieving Cronbach's alpha of SN and PBC to 0.66 and 0.60, respectively. The background details of participants and practices are reported in Table 2.

**Table 2.** Participants and Practice’s Characteristics ^a^

Characteristics	Results	
	Values ^a^	Range
**Female Gender (n = 174)**	116 ± 66.7	
**Age (n = 173)**	32.4 ± 8.1	25-65
**Years of experience as a general practitioner (n = 170)**	5.4 ± 5.8	1-35
**Months of experience as a family physician (n = 165)**	31.1 ± 29.7	2-87
**Daily list size (of consultations) (n = 170)**	41.9 ± 22.1	4-130
**Past referral rate (n=138)**	9.2 ± 5.4	2-33%
**Practice size (n = 162)**		
Solo practice	99 (61)	-
Two-person practice	53 (33)	-
Three-person practice	10 (6)	-
**province (n = 174)**		
M province	91%	-
L province	83%	-

^a^ Data are presented in Mean ± SD or No. (%).

The overall mean scores of the predictor and outcome variables are presented in Table 3. The participants' intention towards the reduction of patient referrals was moderate (mean score of 4.1 on a 1-7 scale). The predictive variables of ATT, SN, and PBC achieved moderate mean scores, too. Investigating the relevant beliefs of indirect variables of TPB revealed that the most strong beliefs expressed by participants included all four control beliefs except one (control belief of perceived "inadequate knowledge and skill of FPs to manage all the patients") in addition to one normative belief of "Formal referents including Health Insurance Organization and District Health Centre authorities".

**Table 3.** Descriptive Statistics of the Theory of Planned Behavior Variables, and the Individual Belief Items ^a^

Variables	Beliefs	Results
**Direct Attitude ** ^**b**^	-	4.27 ± 0.94
**Direct subjective norms ** ^**b**^	-	3.96 ± 1.48
**Direct perceived behavioral control a**	-	4.34 ± 1.12
**Indirect attitude ** ^**b**^ ** (Behavioral beliefs: Bb)**	-	15.89 ± 7.83
	Bb1: better treatment (BB1×OE1) ^c^	16.88 ± 9.46
	Bb2: shorter treatment period (BB2×OE2) ^c^	13.00 ± 8.49
	Bb3: Early diagnosis(BB3×OE3) ^c^	17.85 ± 11.73
	Bb4: patient satisfaction (BB4×OE4) ^c^	15.82 ± 10.75
**Indirect subjective norms ** ^**d**^ ** (normative beliefs : Nb)**	-	11.99 ± 5.42
	Nb1: referents of medical specialists (NB1× MC1) ^e^	13.05 ± 9.01
	Nb2: Referents of patients (NB2× MC2) ^e^	9.93 ± 8.21
	Nb3: referents of Primary care centers' staff (NB3× MC3) ^e^	4.20 ± 4.39
	Nb4: referents of other family physicians (NB4× MC4) ^e^	12.38 ± 10.62
	Nb5: referents of formal authorities including Health Insurance Organization and District Health Centre authorities (NB5× MC5) ^e^	20.38 ± 12.88
**Indirect perceived behavioral control ** ^**d**^ ** (control beliefs: Cb)**	-	18.46 ± 8.15
	Cb1: the patient's insistence to be referred (CB1 × P1) ^f^	20.91 ± 13.12
	Cb2: The constraint of resources (CB2 × P2) ^f^	20.72 ± 11.90
	Cb3: inadequate knowledge and skill of family physician to manage all the patients (CB3 × P3) ^f^	11.19 ± 9.57
	Cb4: the pressures of patients in the form of being rude when they are not referred by family physicians (CB4 × P4) ^f^	21.03 ± 14.97

^a^ Data are presented in Mean ± SD.

^b^ The scores are based on a 1-7 scale.

^c^ Composite scale of behavioral beliefs (BB) × outcome evaluations (OE).

^d^ The composite scores range from 1-49.

^e^ Composite scale of normative belief (NB) × motivation to comply (MC).

^f^ Composite scale of control beliefs (CB)× power to inhibit/facilitate (P).

### 4.1. Predicting Behavioral Intention

The correlation between the intention and direct and indirect variables is showed in Table 4. On regression analysis, the direct variables of ATT, SN, and PBC accounted for 35% of the variance in behavioral intention (Table 5). The strongest predictors were PBC (beta = 0.45, P < 0.001) and SN (beta = 0.35, P < 0.001), indicating that those FPs who perceived a high level of external control factors and social pressure to reduce the referral rate of patients also had more positive intention to decrease the number of patients to medical specialists. Using stepwise method, the background variables were entered into the regression equation model; the factors that could significantly explain a proportion of intention were the practice size and past referral rate behavior. They added further 5% to the prediction of the variance in intention (Table 5).

**Table 4.** Correlations Between Theory of Planned Behavior Variables Across all Respondents

Variables	Intention	Direct Attitude	Direct Subjective Norms	Direct Perceived Behavior Control	Indirect Attitude	Indirect Subjective Norms	Indirect Perceived Behavior Control
**Intention**	-	-	-	-	-	-	-
**Direct attitude**	-0.12	-	-	-	-	-	
**Direct subjective norms**	0.38 ^a^	0.10	-	-	-	-	-
**Direct perceived behavior control**	0.43 ^a^	0.06	-0.02	-	-	-	-
**Indirect attitude**	0.03	0.38 ^a^	0.23 ^a^	-0.07	-	-	-
**Indirect subjective norms**	0.29 ^a^	0.11	0.39 ^a^	-.00	0.33 ^a^	-	-
**Indirect perceived behavior control**	0.11	0.13	0.26 ^a^	-0.18 ^b^	0.43 ^a^	0.37 ^a^	-

^a^ P value < 0.01.

^b^ P value < 0.05.

**Table 5.** Regression Models for Explaining the Variation in Intention to Decrease the Referral Rate

Predictive Variable	B Coefficient (95%CI)	Beta	P Value for B	Model's F Value (p Value)	Model's Adjusted R Square
**Model 1: with TPB ** ^**a**^ ** direct variables**	-	-	-	32.42 (< 0.001)	0.35
Constant	1.45 (0.43-2.47)	-	< 0.01	-	-
Direct perceived behavior control	0.53 (0.39-0.68)	0.45	< 0.001	-	-
Direct subjective norms	0.37 (0.26 -0 0.47)	0.40	< 0.001	-	-
Direct attitude	-0.26 (-0.43-0.08)	-0.18	< 0.01	-	-
**Model 2: With TPB direct variables and background variables ** ^**a**^	-	-	-	17.59 (< 0.001)	0.40
Constant	1.31 (0.11-2.47)	-	< 0.05	-	-
Direct subjective norms	0.40 (0.28-0.53)	0.45	< 0.001	-	-
Direct perceived behavior control	0.43 (0.28-0.59)	0.39	< 0.001	-	-
Direct attitude	-0.20 (-0.40-0.01)	-0.14	< 0.05	-	-
Practice size	0.40 (0.09-0 .72)	0.18	< 0.05	-	-
Past referral rate (PRR)	-0.04 (-0.08-0.01)	-0.17	< 0.05	-	-
**Model 3: With TPB indirect variables**	-	-	-	16.26 (< 0.001)	0.08
Constant	3.24 (2.77-3.71)		< 0.001	-	-
Indirect subjective norms	.07 (0.04 - 0.11)	0.29	< 0.001	-	-
**Model 4: With TPB indirect variables and background variables**	-	-	-	11.57 (< 0.001)	0.15
Constant	2.41 (1.66-3.15)	-	< 0.001	-	-
Indirect subjective norms	0.08 (0.04-0.12)	0.31	< 0.001	-	-
Practice size	0.55 (0.18- 0.92)	0.24	< 0.01	-	-

^a^ The background variables included: age, sex, respondents’ experience as GP and as family physician, average daily list size of consultations, practice size, and past referral rate.

When indirect measures replaced the direct measures in the regression equation, the distinct significant predictor of intention was the normative beliefs (indirect subjective norms) that accounted for 8% of variance in intention. Adding background variables improved the strength of the prediction of intention up to 15% through normative beliefs and the practice size (Table 5). Comparing the association between intention and two background variables of practice size and past referral behavior of respondents revealed that they correlated with intention positively (r = 0.214) and negatively (r = -0.123), respectively. The mean intention score among participants who were working in solo practices was 3.91 that was significantly different from others working in practices with two or three FPs (mean = 4. 42 and 4.80 respectively; F = 3.84; P < 0.05).

## 5. Discussion

The overall intention of Family Physicians towards reduction of patient referrals was moderate that was in concordance with achieved mean scores of predictors including ATT, SN, and PBC. We demonstrated that the TPB variables could properly predict the FPs' intention to reduce the referral rate of patients with respiratory diseases to the medical specialists. However, the amount of the predicted variation in intention (R squared= 35%) is smaller than that was reported by Godin et al. (18) for clinical behavior among physicians in 24 studies (Frequency-weighted mean, R squared = 51%). The stepwise regression analysis showed that the main variables explaining the intention to reduce the rate of referrals were PBC and SN, consecutively. In other words, respondents who expressed a higher level of control to manage the referrals showed more intention to decrease the number of referrals and those who felt under more social pressure to reduce the rate of referral patients had higher intention to reduce the referral rate of patients with respiratory diseases to medical specialists. This finding, regardless of the different clinical behavior being studied, was congruent with some studies (23) and inconsistent with others (24-26), in term of predominant predictive variables.

Investigating the contribution of PBC and its corresponding beliefs in affecting the intention of FPs towards reducing the referral rates revealed that the participants, who perceived more control on managing the referral decisions of outpatients, expressed more intention. On the other hand, the main perceived control beliefs were the pressures from patients and constraints of resources in PCCs. The participants believed that once patients insisted on being referred to the higher level but they avoided doing so, it might negatively affect the doctor-patient relationship (27). In this situation, some FPs felt that they had lower control on referral decisions and thus it might adversely influence their decisions to reduce the referrals in the future. These imposed referrals could result in “dumping referrals”, in which a FP aims to relieve pressure on him/herself (28). Both direct and indirect measures of subjective norms independently accounted for a modest amount of variation in intention of FPs to reduce the referral rates. The participants felt under social pressures to reduce the referrals mostly from formal referents including Health Insurance Organization and District Health Centre authorities. In other words, the expectations of two cited referent groups for reducing the referral rates were taken into account by FPs. This indicated that the employed financial mechanisms by purchaser (i.e. Health Insurance Organization) to limit the referrals of patients to medical specialists might affect the FPs' referring decisions. The participants' past referral behavior accounted for a small part of the variation in their intention to reduce the referral rate of patients. This supports the hypothesized theoretical framework, which suppose the past behavior as an effective factor to predict intention (18). Regarding past behavior, the respondents with high rate of referrals (in the month before the survey) expressed lower intention to reduce the rate of patients referred to the medical specialists.

Another background variable that contributed to explain the variation in intention was practice size. This variable positively correlated with intention of FPs to reduce the referral rate of patients to the medical specialists. In other words, the participants working in group practices (i.e. The PCCs with two or three FPs) expressed higher intention than the FPs working in solo practices. It seems some characteristics of practice might affect the intention of FPs on referral decisions of patients that can be investigated in another study. The possible limitation of this study was that the participants were recruited from the two provinces and this might limit generalization of the results. A high response rate in this study was a strength compared to the relatively low response rate reported in the previous studies (19, 29).

The TPB could be a useful theory to understanding the clinical decision-making regarding the referral of patients with respiratory diseases to the medical specialists. The behavioral intention of Iranian FPs to reduce the referrals is mainly driven by PBC as well as normative considerations. These findings contribute to a better understanding of referral decisions by FPs and are of great value in developing interventions to reduce the variation in the referral rate of patients to medical specialists at primary care health centers.

## Appendix 1

Selecting the topic of "respiratory diseases".

First, we conducted a survey to collect the data about kind of diseases (or main complaints) of patients who were referred by FPs to the medical specialists during a three-month period (from September through November 2012). The data gathered from 30 randomly selected rural primary care centers. The most prevalent diseases in referrals were classified in four ICD 10 groups (30) as following: the circulatory system, digestive system, respiratory system, and pregnancy, childbirth, and the puerperium.

In the second stage, we organized a discussion group with 20 FPs from one of the studied regions. The participants believed that in many situations they referred patients only to follow the request of medical specialists (e.g. for periodical visit of patients with chronic diseases such as cardiovascular and diabetics) or to follow the health system requirements (e.g. periodic referrals of the pregnant women to the gynecologists). They, naming these cases as compulsory referrals, believed that a significant number of referred patients with diseases of "circulatory system" and "pregnancy, childbirth, and the puerperium" were placed in this category. In viewpoint of participants, there a kind of uncertainty in clinical diagnosis of some patients with "digestive system" made them to refer the patients to a higher level of care. Reasoning the mentioned features by participants, we excluded the three groups of diseases and the respiratory system diseases was selected as the topic used in this study.
